# Plasma Protein Biomarkers of Hepatocellular Carcinoma in HCV-Infected Alcoholic Patients with Cirrhosis

**DOI:** 10.1371/journal.pone.0118527

**Published:** 2015-03-19

**Authors:** Gustavo Ferrín, Manuel Rodríguez-Perálvarez, Patricia Aguilar-Melero, Isidora Ranchal, Camilo Llamoza, Clara I. Linares, Sandra González-Rubio, Jordi Muntané, Javier Briceño, Pedro López-Cillero, José Luis Montero-Álvarez, Manuel de la Mata

**Affiliations:** 1 Maimónides Institute for Biomedical Research in Córdoba (IMBIC), Reina Sofía University Hospital, Córdoba, Spain; 2 Biomedical Research Centre Network, Digestive and Liver Diseases (CIBERehd), Córdoba, Spain; 3 Hepatology and Liver Transplantation Unit, Reina Sofía University Hospital, Córdoba, Spain; CRCL-INSERM, FRANCE

## Abstract

Hepatocellular carcinoma (HCC) is one of the most common and lethal cancers in the world, with limited options for treatment unless timely diagnosed. Chronic hepatitis C virus (HCV) infection and persistent heavy alcohol consumption are independent risk factors for HCC development, which may induce a specific protein expression pattern different from those caused separately. The aim of the study was to identify protein biomarkers for the detection of HCC in HCV-infected alcoholic patients with cirrhosis in order to improve survival. We compared protein expression profiles of plasma samples from 52 HCV-infected alcoholic patients with and without HCC, using 2-D DIGE coupled with MALDI-TOF/TOF mass spectrometry. The 2-D DIGE results were analyzed statistically using Decyder software, and verified by western-blot and ELISA. In plasma samples from HCV-infected alcoholic patients, we found significantly differential expression profiles of carboxypeptidase-N, ceruloplasmin (CP), complement component 4a (C4a), fibrinogen-alpha (FGA), immunoglobulin mu chain C region, serum albumin, and serum paraoxonase/arylesterase 1 (PON1). Deregulation of plasma/serum levels of the identified proteins was associated to HCV, ethanol consumption, and/or HCC progression. In the validation through ELISA, C4a serum concentration was increased in HCC patients (2.4±1 ng/mg vs 1.8±0.6 ng/mg; p = 0.029), being the only independent predictor of HCC in the multivariate analysis (OR = 2.15; p = 0.015), with an AUROC = 0.70. The combination of C4a, FGA, CP and PON1 improved slightly the predictive ability of C4a alone (AUROC 0.81). In conclusion, we identified proteins related to acute-phase response, oxidative stress, or immune response, whose differential expression in plasma may be attributed to the presence of HCC. Among them, C4a, and its combination with CP, FGA and PON1, could be considered as potentially reliable biomarkers for the detection of HCC in HCV-infected alcoholic patients.

## Introduction

Liver cancer is the sixth most common cancer and the third cause of cancer-related death. Hepatocellular carcinoma (HCC) represents more than 90% of primary liver cancers and its incidence is rising worldwide, being a major global health problem [1]. Chronic hepatitis C virus (HCV) infection is a major cause of liver disease, cirrhosis, and hepatocellular carcinoma (HCC) in alcoholic patients [2]. Chronic HCV infection and persistent heavy alcohol consumption are both independent and significant risk factors for the development of end-stage liver disease and progression to HCC, which act synergistically to accelerate liver injury. Thus, HCV-infected alcoholic patients have been observed to have more rapid and frequent occurrence of fibrosis and cirrhosis as compared with non-alcoholic liver patients. Some of the major postulated mechanisms responsible for disease progression include high rates of apoptosis, lipid peroxidation, and generation of free radicals and reactive oxygen species with reduced antioxidant capacity of the liver [3].

HCC develops in a cirrhotic background in up to 90% of cases and is frequently identified at advanced stages of disease when its prognosis is rather poor. Curative therapies are available only for early tumors, most of them unfortunately asymptomatics [4]. Tests HCC screening include serological and imaging examinations. Ultrasonography is the most widely used imaging test for screening because of its acceptable diagnostic accuracy, non-invasiveness, good acceptance by patients and moderate cost. However, fibrosis septa and regenerative nodules present in liver cirrhosis may hinder the identification of small tumors by this technique, which is highly dependent on the operator’s experience. Among serological biomarkers, alpha-fetoprotein is the most extended serum marker for HCC screening, but it is not routinely used by clinicians due to its insufficient sensitivity and specificity [1,5]. While we await the development of new surveillance strategies for high-risk patients and real curative treatments for advanced HCC, identification of biomarkers for early detection of HCC is essential to improve patient survival.

Two-dimensional fluorescence difference gel electrophoresis (2-D DIGE) analysis of clinical samples has been widely used in combination with mass spectrometry (MS) to identify potential biomarkers for the detection and diagnosis of human cancer, as well as to understand mechanisms involved in the process of carcinogenesis [6]. In addition, available depletion strategies have demonstrated effective removal of high abundance proteins and improvement in the detection of less abundant serum/plasma proteins [7]. The aim of this pilot study was to identify new protein biomarkers of HCC by comparing protein expression profiles of plasma samples from HCV-infected alcoholic patients with and without HCC using 2-D DIGE coupled to MALDI-TOF/TOF MS. As noted, ethanol consumption and chronic HCV infection are independent risk factors for HCC development, and also able to modulate gene expression and protein synthesis in the liver, leading to a specific expression pattern. In addition, these two factors can synergize to accelerate liver injury. Thus, the coexistence of both factors, ethanol and HCV, could alter the expression pattern that is caused independently by any of them during the development of hepatic disease.

## Materials and Methods

### 1. Patients and plasma samples

From a prospectively collected database of patients with end stage liver disease at the Reina Sofía University Hospital, Córdoba, Spain, (2005–2013) we consecutively selected 52 patients with abusive alcohol intake (defined as regular daily ethanol consumption superior to 40 g) and HCV infection. Among them 25 patients had HCC (tumor group) and 27 patients were HCC free (control group). From each group 3 patients paired for age, sex and Child-Pugh were selected for DIGE analysis, and the remaining patients served as a validation cohort (by using Western Blot and ELISA). There were no exclusion criteria for the present study. We included patients with different tumor characteristic in order to identify biomarkers of HCC regardless of the tumor progression.

All patients showed F4 fibrosis, either by liver biopsy or transient elastometry (Fibroscan). The diagnosis of HCC was confirmed by imaging techniques (computed axial tomography or magnetic resonance imaging) on the basis of non-invasive criteria (EASL-EORTC clinical practice guidelines) [1]. Basal characteristics of included patients are shown in Table 1. Blood samples were collected according to our protocol in sterile tubes with K3-EDTA anticoagulant (9 ml K3E VACUETTE, Greiner Bio-One, Austria), and plasma was separated by centrifugation at 2900 g for 5 min at 4° C. Aliquots of plasma (500 μl) were immediately taken and stored at—80° C until analysis. Samples were subsequently transferred to the Andalusian Public Health System Biobank (BBSSPA) from the Reina Sofía University Hospital. Written informed consent to participate in this study was obtained from each patient and approval to conduct this study was granted by the Reina Sofía University Hospital Ethics Committee.

**Table 1:** Clinical information of patients included in DIGE analysis and ELISA.

	DIGE analysis		ELISA validation		
VARIABLE	HCC	CONTROL	HCC	CONTROL	
	(n = 3)	(n = 3)	(n = 22)	(n = 24)	*p*
Age	48.3±3.5	44.7±4.0	62.5±11.7	50.0±12.7	0.001
Gender; n (%)					
Male	3 (100%)	3 (100%)	21 (95.5%)	24 (100%)	0.47
Female	0 (0%)	0 (0%)	1 (4.5%)	0 (0%)	
Child-Pugh; n (%)					
A	1 (33.3%)	1 (33.3%)	13 (59.1%)	5 (20.8%)	0.008
B-C	2 (66.7%)	2 (66.7%)	9 (40.9%)	19 (79.2%)	
MELD	12.3±3.2	15.3±4.0	12.3±5.8	15.5±5.9	0.072
AFP* (ng/ml)	17.0±15.6	4.4±3.8	32.9	4.8	0.050
			(IQR 5.3–258.6)	(IQR 2.1–8.4)	
Number of HCC nodules	1.3±0.6	-	1.77±1	-	-
Number of HCC nodules					
Uninodular	2 (66.7%)	-	11 (50%)	-	-
Multinodular	1 (33.3%)		11 (50%)		
Diameter of main nodule (mm)	31.2±14.7	-	37.7±20	-	-

Data expressed as mean ± standard deviation; *p*-values from univariate analysis are included for the validation cohort.

### 2. Removal of high-abundance proteins from plasma samples

Plasma samples were processed using the ProteoMiner Protein Enrichment Kit (BioRad, Hercules, California, U.S.A), which removes high-abundance proteins and makes possible the detection of medium- and low-abundance proteins. Samples were processed according to the manufacter’s recommendations using an initial amount of protein of 10 mg in a volume of 200 μl. Samples were subsequently cleaned prior to labelling using a 2-D Clean-Up Kit (GE Healthcare, Piscataway, U.S.A). The protein pellets were resuspended in ice-cold lysis buffer (30 mM Tris, 2 M Tiourea, 7 M Urea, 4% w/v CHAPS) and pH adjusted between 8.5–9 with 0.5 M NaOH. Protein quantification was performed using the 2-D Quant Kit (GE Healthcare).

### 3. DIGE labelling, protein separation and gel imaging

Samples were labelled with CyDye DIGE Fluors (GE Healthcare). Briefly, 50 μg of depleted protein samples from control and tumor groups were alternately labelled with Cy3 and Cy5 for comparison on the same 2-D gel. It was also prepared a pool of all samples labelled with Cy2 to be used as a standard in all gels to aid image matching and cross-gel statistical analysis. The Cy3 and Cy5 labelling reactions (50 μg of each one) from each lysate and an equal amount of Cy2-labelled standard were mixed and run in the same 2-D gel. Isoelectric focusing protocol and 2-D electrophoresis conditions are summarizes in [8]. The Cy2, Cy3 and Cy5 channels for each gel were individually imaged on a Typhoon 9400 Variable Mode Imager (GE Healthcare). Statistics and quantification of protein expression for spot selection were performed with *Decyder* software (GE Healthcare).

### 4. Spot digestion and MS analysis

The spots digestion and MALDI-TOF analysis was carried out in the UCO-SCAI proteomics facility (Córdoba, Spain. Carlos III Networked Proteomics Platform, ProteoRed-ISCIII). The selected protein spots were automatically picked from the gel and the resultant gel fragments were digested with trypsin and analysed by MALDI-TOF/TOF MS. Protein identification was carried out by combination of the MS spectra and their MS/MS with the NCBInr database, using MASCOT (MatrixScience) as a searching engine. The steps and conditions that were carried out are summarized in Ferrín et al, 2013 [8].

### 5. Western Blot analysis

Western Blot analysis was only performed as a control of DIGE results and MS identification. We selected the differentially expressed proteins carboxypeptidase N (CPN), paraoxonase 1 (PON1) and ceruloplasmin (CP). 10 μg of protein from processed plasma samples were separated on 12% SDS-PAGE, transferred to nitrocellulose membranes (PALL Life Sciences, New York, U.S.A), and incubated with 1:200 diluted primary antibodies against CPN (goat polyclonal antibody; sc-18966), PON1 (rabbit polyclonal antibody; sc-133919), and CP (mouse monoclonal antibody; sc-365205) (Santa Cruz Biotechnology, California, U.S.A). Immunodetection was visualized by chemiluminescence, using ECL Advance (GE Healthcare) and the LAS-3000 Imager instrument (Fujifilm).

### 6. Enzyme-linked immunosorbent assay (ELISA)

ELISA tests were used to quantify the protein concentration of differentially expressed proteins CPN, CP, complement component 4a (C4a), fibrinogen-alpha (FGA), PON1 (USCN Life Science Inc., Wuhan, China), and immunoglobulin M (IgM) (Abcam, Cambridge, UK) in plasma samples from the control (24 cases) and tumor (22 cases) groups. The assays were carried out following the manufacturer’s instruction, and the obtained data were used to evaluate the sensitivity and specificity of the biomarker in the diagnosis of HCC in HCV-infected alcoholic patients with cirrhosis.

### 7. Statistical analysis

Variables were displayed in frequency tables or expressed as means and standard deviations, or as medians and interquartile range (IQR) for those with asymmetric distribution. Chi square test was used for frequencies, student’s T or ANOVA tests for quantitative variables, and Mann-Whitney’s U or Kruskal-Wallis for asymmetric distributions. The optimal threshold value of candidate biomarkers for identifying patients with HCC was established by receiver operating characteristic (ROC) curves choosing the lowest threshold possible with the highest negative predictive value. Multiple logistic regression was used to explore whether a combination of multiple biomarkers is able to improve the predictive accuracy. A *p*-value of less than 0.05 was considered statistically significant.

## Results

From 52 patients enrolled, 3 patients with HCC and 3 patients without HCC were randomly selected for the DIGE analysis. The features of these patients are described in table 1 (on the left). The remaining patients served as the validation cohort including 22 patients in the HCC group, and 24 patients in the control group (table 1 on the right). Among the validation cohort HCC patients were older and showed better liver function than patients without HCC. Indeed, 13 out 22 patients with HCC (59.1%), and only 5 out 24 patients from the control group (20.8%) were Child A (p = 0.008). The MELD score was also marginally reduced in patients with HCC (12.3±5.8 vs 15.5±5.9; p = 0.072). Mean age of HCC patents was 62.5±11.7 compared to 50±12.7 in controls (p = 0.001). Thus both age and liver function were considered as potential confounding factors, and were controlled in the multivariate logistic regression analysis as described below. In the HCC group the mean number of nodules was 1.77±1, having 50% of patients uninodular HCC. The diameter of the main nodule was 37.7±20 mm.

### Analysis and identification of differentially expressed proteins

**Fig 1:**
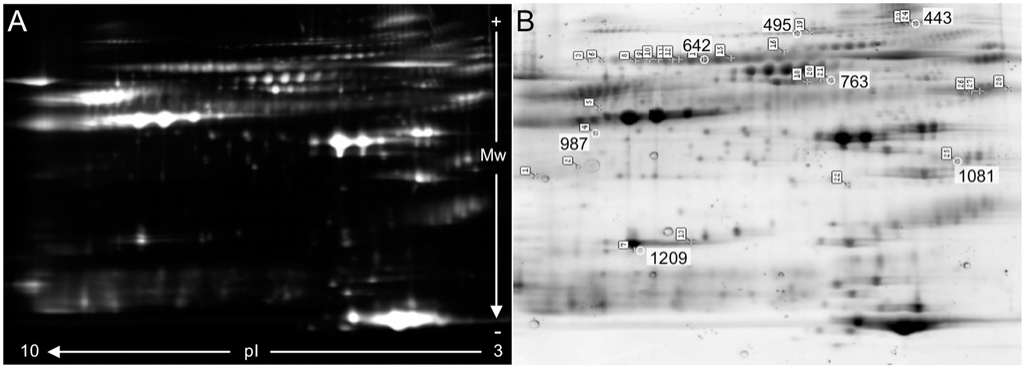
2-D gel images. (A) Overlay master gel image containing Cy2-, Cy3- and Cy5-labeled proteins of plasma samples. (B) SYPRO Ruby (BioRad) stained preparative gel image. Differentially expressed protein spots identified are marked.

After 2-D DIGE, among matched protein spots, 19 spots were differentially regulated in the HCC group in a statistically significant way. Out of the 19 differentially expressed spots detected by DIGE analysis, 7 different proteins were identified by MALDI-TOF/TOF MS as carboxypeptidase-N (CPN), ceruloplasmin (CP), complement component 4a (C4a), fibrinogen-alpha (FGA), immunoglobulin mu chain C region (IgM), serum albumin, and serum paraoxonase /arylesterase 1 (PON1). Differentially expressed spots and their fold change expression in HCC versus control samples are shown in Fig. 1 and Table 2.

**Table 2:** Differentially expressed proteins identified in plasma samples.

Spot no.	*p*-Value	Fold change	Protein name	Accession No	Protein score	Peptide count	Protein function	Classification
598	0.023	1.88	Immunoglobulin M chain C region	gi:4467842	187	15	Antigen binding	Immune response
625	0.049	2.33		gi:7439150	545	19		
627	0.039	1.88		gi:41388180	553	19		
633	0.027	2.09		gi:41388180	560	19		
634	0.050	1.84		gi:7439150	460	17		
641	0.041	1.80		gi:7439150	538	18		
642	0.031	1.81		gi:7439150	582	19		
987	0.044	1.67	Carboxypeptidase-N	gi:146386938	155	9	Protease	Defence response
1081	0.040	1.78	Serum paraoxonase/ arylesterase 1	gi:190194	238	9	Hydrolase	
495	0.020	1.86	Fibrinogen-alpha	gi:237823914	385	23	Haemostasis, platelet activation, extracellular matrix	Acute-Phase Response
899	0.049	1.43		gi:237823914	568	27		
1091	0.041	1.47		gi:237823914	265	16		
443	0.014	1.50	Ceruloplasmin	gi:1620909	494	23	Iron/copper homeostasis	
451	0.037	1.51		gi:221042622	660	28		
1186	0.050	1.98	Complement component 4a	gi:34782950	569	14	Complement component	
1209	0.027	1.86		gi:34782950	375	12		
762	0.038	2.02	Serum Albumin	gi:31615331	464	28	Protein carrier	Regulation of the colloidal osmotic pressure of blood
763	0.025	2.45		gi:168988718	205	13		
765	0.037	2.05		gi:168988718	835	29		

Statistical significance determined by *t*-test, where *p* ≤ 0.05.

### Confirmation of DIGE-results by western blotting

**Fig 2:**
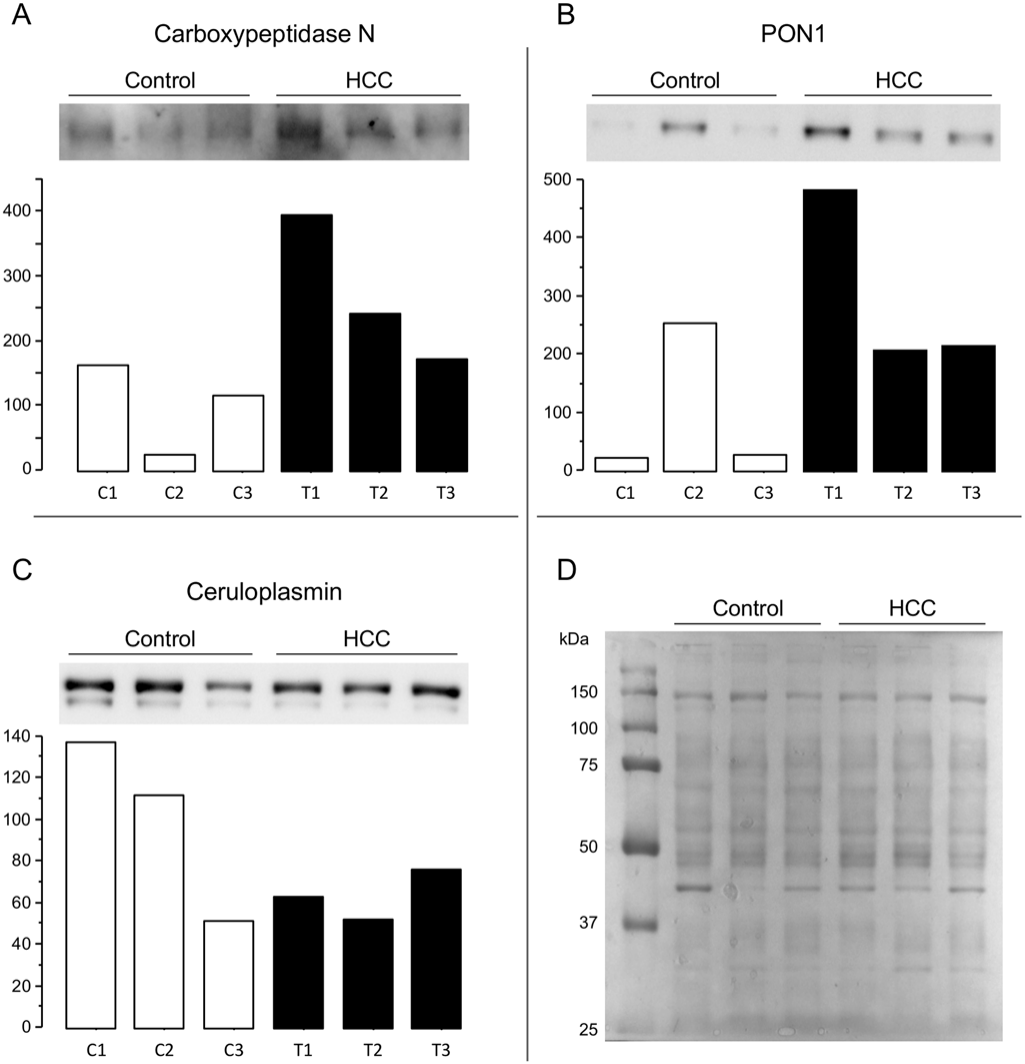
Confirmation of DIGE results by western-blot. Detection and quantification of (A) carboxypeptidase-N (CPN), (B) serum paraoxonase /arylesterase 1 (PON1), and (C) ceruloplasmin (CP) in depleted human plasma samples previously analysed by DIGE. Protein band density was calculated by *QuantityOne 4*.*4*.*0* (BioRad) software. Results are shown as arbitrary units (AU); (D) Transferred proteins to the nitrocellulose membrane were detected by Ponceau S stain as western-blot loading control. C1, C2 and C3: protein samples of patients from the control group; T1, T2 and T3: protein samples of patients from the tumor group.

In order to verify DIGE/MS results, we randomly chose three proteins from those previously identified by MS. Thus, we confirmed the relative expression plasma level changes for the identified proteins CPN, CP, and PON1 by western-blot (Fig. 2).

### Validation of identified proteins as candidate biomarkers for HCC development

**Fig 3:**
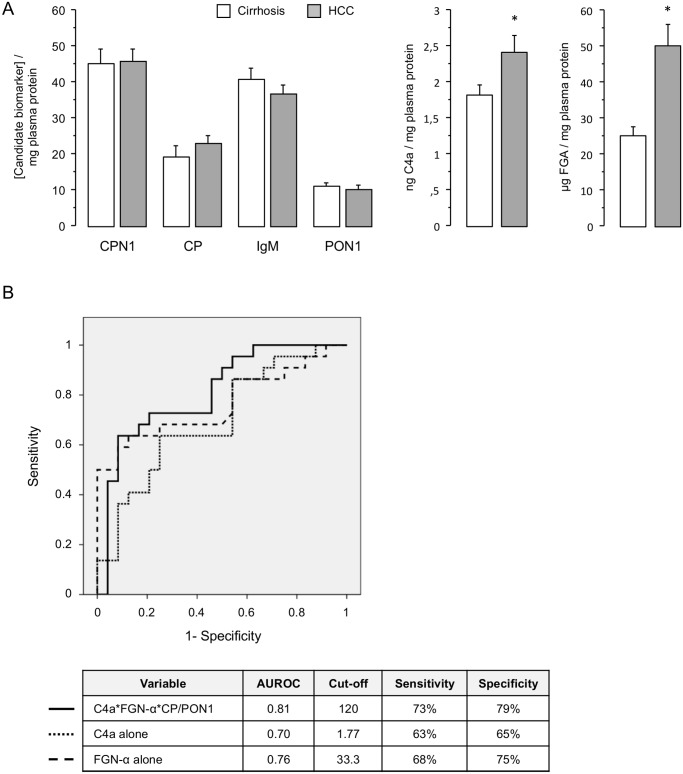
Validation of selected candidate biomarkers for HCC development in a larger patient population. (A) Quantification of plasma concentration of CPN (pg/mg plasma protein), CP, IgM, PON1 (μg/mg plasma protein), C4a (ng/mg plasma protein) and FGA (μg/mg plasma protein) by ELISA. The sample size was 24 in the control group and 22 in the tumor group. * Statistically significant differences between groups. Data as mean ± standard error; (B) ROC curves showing that the best predictive ability to identify patients with HCC belongs to the combination of C4a, FGA, CP and PON1.

To evaluate the utility of the identified proteins CPN, CP, C4a, FGA, IgM and PON1 as candidate biomarkers for HCC development in HCV-infected alcoholic patients, 46 plasma samples (24 from the control group and 22 from the tumor group) were subsequently analyzed by ELISA (Fig. 3A). The immunoassay confirmed the differential expression of C4a in HCC patients (2.4±1 ng/mg plasma protein) compared to patients without HCC (1.8±0.6 ng/mg) (p = 0.029). Average concentrations of FGA were increased in HCC patients (49.9±28 vs 25±12 μg/mg) (p<0.001). Although there were higher concentrations of CP in HCC patients (21.8 μg/mg [IQR 14–27]) than in controls (16.1 μg/mg [IQR 11–24]), these differences were marginally not significant (p = 0.071). Serum PON-1 concentration was 8.3 μg/mg [IQR 6–13] in HCC patients and 11.5 μg/mg [IQR 7–13] in controls (p = 0.31). The serum concentration of CPN was similar in HCC patients compared with controls without HCC (45.5±16.5 ng/mg vs. 44.9±20.1 ng/mg; p = 0.91), as it was IgM concentration (36.7±11 vs 40.5±15.5; p = 0.34).

In the multivariate logistic regression analysis C4a emerged as the only independent predictive factor of HCC (OR = 2.15; 95% CI = 1.16–4.10; p = 0.015), whereas plasma fibrinogen was marginally not significant (p = 0.058) (Table 3). The area under ROC curve for C4a was 0.70. A cut-off of 1.77 ng of C4a /mg plasma protein represented an appropriate value to distinguish cirrhotic patients from HCC patients, with a sensitivity of 63% and a specificity of 65%. However the best predictive ability was found with the combination of C4a, FGA, CP and PON1 through the following formula:

$$\frac{C4a\times FGA\times CP}{PON1}$$

The area under ROC curve of this combination to identify patients with HCC was 0.81, which was higher than the area under ROC curve of C4a and FGA separately (Fig. 3B). A threshold of 120 for the combination of C4a, FGA, CP and PON1 had a sensitivity of 73% and a specificity of 79%.

**Table 3:** Multivariate logistic regression analysis for the candidate biomarkers.

VARIABLE	OR	95% CI	*p*
C4a*	2.15	1.16–4.10	0.015
VARIABLES ELIMINATED FROM THE MODEL			
CPN	1	0.92–1.10	0.86
PON-1	0.98	0.77–1.23	0.85
CP	1.03	0.97–1.10	0.23
FGA	1.05	0.99–1.13	0.058
IgM	0.94	0.85–1.05	0.33
VARIABLES CONTROLLED AS POSSIBLE CONFOUNDING FACTORS			
Age	1.1	1.02–1.17	0.01
CHILD (A vs. B-C)	0.14	0.15–1.39	0.09
MELD	1	0.85–1.18	0.95

Complement component 4a was the only independent predictor of HCC after controlling for possible confounding factors.

*OR and confidence interval for C4a has been calculated for an increment of 0.5 ng/mg plasma protein.

C4a: Complement component 4a; CPN: carboxypeptidase-N; PON1: serum paraoxonase /arylesterase 1; CP: ceruloplasmin; FGA: fibrinogen-alpha; IgM: immunoglobulin mu chain C region.

The serum concentration of C4a was significantly increased in patients with multinodular HCC (3.05±1.17 ng/ml) when compared with patients with uninodular HCC (1.75±0.74 ng/ml) (p = 0.004). The remaining biomarkers, including the combination of C4a, FGA, CP and PON1 through the proposed formula, were not related to the number of HCC nodules. None of them (C4a included) correlated with the size of the largest nodule.

## Discussion

DIGE analysis coupled to MALDI TOF/TOF MS identified seven different proteins involved in biological processes such as the acute phase response, immune/defence response, or cell adhesion. They have been summarized and classified according to their function in Table 2. Of these, serum albumin was not considered for further analysis because its concentration in plasma is a clear indicator of liver function, and impaired liver function could deprive it of its potential role as a biomarker for HCC.

CP and PON1, both liver-synthesized glycoproteins with antioxidant properties [9,10], were identified in plasma of HCC patients as deregulated proteins that have been previously related to alcohol consumption [11,12] and/or HCC development [13,14,15]. Free radical generation increase and decreased antioxidant liver ability are considered two main events responsible for disease progression in HCV-infected alcoholics. However, ELISA failed to confirm the results in a larger patient population.

In addition to these proteins, we also identified FGA as an up-regulated protein related to HCC in HCV-infected alcoholic patients. Increased plasma/serum fibrinogen is related to chronic alcohol consumption [16] and liver diseases, and could serve as a useful predictor for clinical progression of HCC [17]. This fact could be explained as part of the acute response and related to malignant-associated inflammation [18], by an increased plasma anti-thrombin activity [19], or by an abnormal fibrinogen synthesis or impaired removal by the diseased liver [20]. As extracellular matrix protein, the deposition of fibrinogen could serve to sequester complement components in tumor areas [21], or as a scaffold to support binding of growth factors and to promote cell adhesion, proliferation, and migration during angiogenesis and tumor cell growth [22]. In the present study, FGA was increased in HCC patients univariately, but it did not behave as an independent predictive factor of HCC in the multivariate logistic regression analysis.

The remaining identified proteins, including CPN, IgM and C4a, are part of or have also been directly related to the complement system. Complement is an integral part of the innate immunity, and consists of a number of small proteins (complement components) mainly synthesized by the liver, which help to antibodies and phagocytic cells to clear pathogens. Complement components normally circulate in the blood as inactive precursors, which can be activated by specific proteases under certain stimuli. This results in an amplifying cascade of further cleavages, with massive amplification of the response and activation of the cell-killing membrane attack complex [23]. Apart from its role in infections, complement also participates in the clearance of immune complexes and apoptotic cells, the anti-cancer defence [24], and the pathogenesis of a variety of liver disorders including liver injury and repair, viral hepatitis, or alcoholic liver disease [25].

IgM is part of the adaptive immune system and plays a key role in the complement activation by causing complement component 3b binding to the antigen as part of the opsonisation process. Elevated levels of secretory immunoglobulins have been demonstrated in several liver dysfunctions such as alcoholic liver disease [26] or HCV infection [27]. In fact, anti-HCV IgM is detected at high rates in patients with chronic HCV [28]. In addition to the involvement on primary defence mechanism, IgM antibodies show a significant anti-cancer activity [29]. Thus, squamous cell carcinoma antigen-IgM complexes have been proposed as biomarkers for HCC [30]. Here, DIGE analysis showed deregulated levels of IgM in plasma samples from patients with HCC. In this patient group, IgM was identified in seven different up-regulated protein spots. However, plasma concentration of IgM was similar in HCC patients and controls without HCC when it was measured by ELISA. This may be due to that the 2-D DIGE analysis detects posttranslational modifications of proteins that may be missed in the ELISA.

CPN is a zinc metalloprotease that has been associated with the complement system by its ability to cleave carboxy-terminal arginines and lysines from peptides found in the bloodstream such as complement anaphylotoxins (i.e. C4a) inactivating them. Thus, it has been suggested that the protease can be protective by preventing the accumulation of potentially harmful levels of such compounds in the circulation [31]. The expression of CPN may be altered by different conditions, including cirrhosis or cancer. While the first one decreases it, certain types of cancer increase plasma CPN levels [32,33]. In the present study, the increased plasma CPN level detected by DIGE analysis could not be confirmed by ELISA in a larger sample population.

C4a is a fragment of the complement component 4 that is produced by proteolysis during the activation of the classical or lectin pathways of the complement system. This peptide is part of the referred to as anaphylatoxins group, which participates in a broad range of biological activities such as vascular permeability, chemotaxis, or inflammation. As mentioned above, alcohol and HCV can regulate the expression of a number of cellular genes, including those related to complement. In experimental models, alcohol and its metabolites are able to activate complement and contribute to the pathogenesis of alcoholic liver disease. However, the precise role of the complement system on the alcoholic liver disease has not yet been determined [25]. Several studies have illustrated the role of HCV proteins on the innate immune system by inhibiting complement components production with functional impairment of the membrane attack complex [34,35,36]. In particular, C4 mRNA levels were reduced in liver tissue samples from patients with chronic HCV infection, in hepatocytes transfected with RNA from HCV, and in HCV core or NS5A transgenic mice [37]. In the presence of HCV proteins, gamma interferon (IFN-g) failed to induce C4 promoter activation and the expression of an important transcription factor for IFN-g-induced C4 expression [38]. On the other hand, it has been shown that transgenic mice that conditionally express intermediate HCV core protein develop inflammation and activation of complement [39]. Complement activation and subsequent deposition of complement components on tumor tissues have been demonstrated in cancer patients, and have been suggested as candidate biomarker for the diagnosis of HCC in patients with cirrhosis [40,41,42,43]. In our study, increased plasma levels of C4a were independently associated to HCC development in HCV-infected alcoholic patients with cirrhosis, and showed a fair diagnostic accuracy (AUROC 0.70). Furthermore, the serum concentrations of C4a were two-fold increased in patients with multinodular HCC when compared with patients with uninodular disease, and thus suggesting that more advanced HCC with increased tumor burden triggers the production of C4a. Therefore, upregulated plasma levels of C4a in HCV-infected patients with HCC may be associated with the immune response to HCV infection, alcohol consumption, and/or tumor antigens [44].

All the herein identified proteins are hepatic synthesized that have been previously related to HCV infection and/or ethanol consumption. In this regard, it is noteworthy that none of the proteins that have been identified in this work matches any of those that we previously identified in the plasma of non-alcoholic HCV-infected patients during the development of HCC [8]. However, the proteins identified in that previous study were involved in similar biological processes.

After controlling for possible confounding factors such as age and liver function, C4a could be considered as a potential biomarker for the detection of HCC in HCV-infected alcoholic patients. Interestingly, the best ability to detect HCC was found when C4a was combined with the proteins identified in DIGE analysis FGA, CP and PON1, through the proposed formula (AUROC 0.81). Thus, a threshold of 120 had a sensitivity of 73% and a specificity of 79%. Further studies, with larger cohorts, are needed to validate these results, and to establish the actual role of a serological test for HCC screening in high-risk population.

Finally, the increased concentrations of C4a found in patients with more advanced HCC reinforce the hypothesis that this may be a reliable biomarker in patients with end stage liver disease related to HCV and alcohol. In this regard, it is noteworthy that complement not only participates in tumor clearance but also promotes tumor growth. It has been shown that C3 or C4 deficiency and complement-activation products inhibition result in greatly reduced tumor growth [45]. Moreover, anaphylatoxin C5a appears to contribute to cancer progression rather than removal [46,47]. Because only biomarkers that directly contribute to tumor growth may be targeted for therapy, the possibility of exploiting C4a as therapeutic target in HCC deserves further investigation in order to determine its role in tumor development.
